# Electroactive Oxidized Alginate/Gelatin/MXene (Ti_3_C_2_T_x_) Composite Hydrogel with Improved Biocompatibility and Self-Healing Property

**DOI:** 10.3390/polym14183908

**Published:** 2022-09-19

**Authors:** Hui Zhu, Weitao Dai, Liming Wang, Cong Yao, Chenxi Wang, Bingsong Gu, Dichen Li, Jiankang He

**Affiliations:** 1 State Key Laboratory for Manufacturing Systems Engineering, Xi’an Jiaotong University, Xi’an 710049, China; hui.zhu@xjtu.edu.cn (H.Z.); david1@stu.xjtu.edu.cn (W.D.); wlm2001@stu.xjtu.edu.cn (L.W.); yaocong19970221@stu.xjtu.edu.cn (C.Y.); wcx-renjv@stu.xjtu.edu.cn (C.W.); gubingsong@stu.xjtu.edu.cn (B.G.); dcli@mail.xjtu.edu.cn (D.L.); 2 NMPA Key Laboratory for Research and Evaluation of Additive Manufacturing Medical Devices, Xi’an Jiaotong University, Xi’an 710049, China

**Keywords:** electrically conductive hydrogel, MXene, oxidized alginate, self-healing, wound healing

## Abstract

Conductive hydrogels (CHs) have shown promising potential applied as wearable or epidermal sensors owing to their mechanical adaptability and similarity to natural tissues. However, it remains a great challenge to develop an integrated hydrogel combining outstanding conductive, self-healing and biocompatible performances with simple approaches. In this work, we propose a “one-pot” strategy to synthesize multifunctional CHs by incorporating two-dimensional (2D) transition metal carbides/nitrides (MXenes) multi-layer nano-flakes as nanofillers into oxidized alginate and gelatin hydrogels to form the composite CHs with various MXene contents. The presence of MXene with abundant surface groups and outstanding conductivity could improve the mechanical property and electroactivity of the composite hydrogels compared to pure oxidized alginate dialdehyde-gelatin (ADA-GEL). MXene-ADA-GELs kept good self-healing properties due to the dynamic imine linkage of the ADA-GEL network and have a promoting effect on mouse fibroblast (NH3T3s) attachment and spreading, which could be a result of the integration of MXenes with stimulating conductivity and hydrophily surface. This study suggests that the electroactive MXene-ADA-GELs can serve as an appealing candidate for skin wound healing and flexible bio-electronics.

## 1. Introduction

Advances in health security and artificial intelligence have flourished in recent years with the development of flexible wearable technologies. Electronic skins (E-skins) have emerged as one of the most potential applications which are able to mimic human skin and serve as a bridge to transduce external stimuli to electrical signals [1,2]. An ideal engineered E-skin system should meet versatile demands such as the adaptability of the machinery of natural skin, sufficient conductivity enabling the communication of users with their surroundings via transmission of an electrical signal, good biocompatibility and self-healing ability for wound repair after skin injury [3]. Those challenging requirements motivate the development of new materials for fabricating E-skins including ionic liquids [4], liquid metals [5] and other two-dimensional materials [6] integrated with stretchable sheets. However, there is still a large need to develop highly compliant and biocompatible materials for E-skins to mimic the functionalities of real skin.

Conductive hydrogels (CHs) have attracted considerable interest as soft electronics with flexible mechanical properties and tunable electrical conductivity, which have shown great potential in next-generation materials for flexible epidermal sensors [7,8]. Furthermore, CHs have an inherent tissue-like nature, i.e., water-rich structure, three-dimensional (3D) network construction and adaptable modulation, which would be beneficial to tissue regeneration [9,10]. So far, conductive hydrogels were mainly prepared by incorporation of intrinsically conductive polymers [11,12], metal particles [13], carbon-based nanofillers [14,15,16], or charged salts [17,18] into the hydrogel matrix. However, the biocompatibility and self-healing properties of current CHs are still limited, and their fabrication methods are normally very complicated. It is, therefore, necessary to engineer CHs capable of self-healing with a dynamic cross-link network and excellent biocompatibility to be well used in E-skin systems.

Transition metal carbides and/or nitrides (MXenes) are a type of two-dimensional (2D) electrochemical material with a layered structure and a variety of terminated functional groups (–O, –OH, and –F) on their surface [19]. The fabulous surface properties allow the formation of MXene-based hybrids by a combination of MXenes with other organic or inorganic materials using self-assembly, polymerization, and covalent interactions [20,21]. In addition, alginate is a natural and anionic polysaccharide widely used in tissue engineering owing to its superior biocompatibility [22,23]. Gelatin is a biomacromolecule that can support cell adhesion due to the presence of arginine glycine-aspartic [24,25]. Recent research has reported the fast self-gelation of alginate aldehyde (ADA) and gelatin (ADA-GEL) relaying on the Schiff’s base reaction without any additional initiator under moderate conditions [26,27]. More importantly, a dynamic network can be formed inside ADA-GEL, which supports better cell proliferation and differentiation [27].

**Figure 1:**
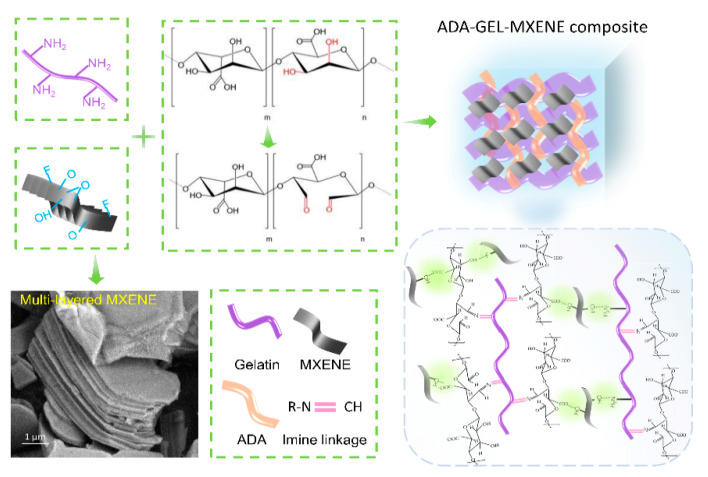
Schematic illustration of the fabrication of composite hydrogel composed of MXene nano-flakes, ADA and gelatin, and the multi-layered structure of MXene nano-flakes.

In this work, we aim to fabricate conductive, self-healing, and biocompatible hydrogels consisting of MXenes, ADA, and gelatin via a “one-pot” strategy (Figure 1) by simply incorporating multi-layered MXene nanosheets with a concentration of 0.2 and 2 *w*/*v*%, respectively, into the ADA-GEL network at room temperature. The effect of MXene addition on hydrogel microstructure, rheology, as well as electroactivity was investigated. In addition, the biological behaviors of mouse embryonic fibroblasts (NH3T3s) on different composite hydrogels were examined in detail. The developed composite hydrogels incorporated with MXenes are expected to provide a suitable electrical and mechanical microenvironment for NH3T3 cells.

## 2. Materials and Methods

### 2.1. Materials

Sodium periodate (NaIO_4_), ethanol (≥99.8%), ethylene glycol, alginate (sodium salt of alginic acid from brown algae, suitable for immobilization of microorganisms, guluronic acid content 65–70%) and gelatin (Bloom 300, Type A, from porcine skin) were purchased from Sigma-Aldrich, Darmstadt, Germany. Calcium chloride (CaCl_2_) was purchased from Macklin, Shanghai, China. Transglutaminase (mTG) was purchased from Bomei Tec. Co, Hefei, China. Dialysis tubes (MWCO: 6–8 kDa) were from Thermo Fisher Scientific^TM^ (Waltham, MA, USA). MXene (Ti_3_C_2_T_x_, Multi-layered nanosheets) were purchased from Xinxi Tec. Co., Guangdong, China. Calcein AM, Rhodamine phalloidin, Hoechst, Dulbecco’s Phosphate Buffered Saline and 2-(4-Amidinophenyl)-6-indolecarbamidine dihydrochloride (DAPI) were obtained from Thermo Fisher ScientificTM (Waltham, MA, USA). Ultrapure water was obtained by Ultrapure (UPR-II, Shenzhen, China).

### 2.2. Preparation of Alginate-Di-Aldehyde (ADA)

Briefly, 10 g of sodium alginate was dispersed in 50 mL of ethanol followed by adding 50 mL of aqueous solution of NaIO_4_ (6.416% *w*/*v*) in the absence of light. After 6 hours of stirring at room temperature (RT), the reaction was quenched by adding 10 mL of ethylene glycol and kept stirring for another 30 min. The ADA products were obtained after dialysis against ultrapure water for 5 days and a one-week freeze-drying procedure (Alpha 1–2 LD plus, Martin Christ, Osterode am Harz, Germany). 

### 2.3. Preparation of ADA-GEL (AG) Hydrogel

ADA and gelatin were dissolved in Dulbecco’s phosphate-buffered saline (DPBS) with a concentration of 2.8 *w*/*v*% and 11.2 *w*/*v*%, respectively. ADA-GEL was prepared by adding ADA solution dropwise to GEL solution to obtain a final polymer concentration of 7.0 *w*/*v*% under continuous stirring and standing in a mold for 10–20 min for gelation. For preparing the composite hydrogels, MXenes were firstly dispersed in DPBS by ultrasonic for 30 min followed by adding ADA to prepare a mixture solution with the ADA content of 2.8 *w*/*v*%. Then, gelatin solution (11.2 *w*/*v*%) was added slowly into the illustrated mixture under stirring and gelation for 10–20 min. The final concentration of MXenes in the composite hydrogels are 0.02 and 0.2 *w*/*v*%, respectively, named ADA-GEL-0.2M and ADA-GEL-2M.

### 2.4. Characterization of Hydrogel

The different hydrogels were freeze-dried and the chemical structure of hydrogels was analyzed by Attenuated Total Reflectance Infrared (ATR-IR) spectroscopy over a wavenumber range of 4000–400 cm^−1^ using the transmission mode (Bruker Alpha, Leipzig, Germany). The microstructures of hydrogels were observed by scanning electron microscopy (SEM, HITACHI Ltd., Tokyo, Japan). The Nyquist curves of different hydrogels were tested on an Autolab PGSTAT204 workstation. The conductivity σ of hydrogels can be estimated by the following equation [28]:

σ
= *L*/*RS* (1)

where *L* and *S* are the thickness and area of the hydrogels and *R* (Ω) represents the bulk resistance determined from the intercept of Nyquist plots with the real axis. The rheological was tested using a rheometer (MALVERN Ltd., Cambridge, UK) at 25 °C. The hydrogels were prepared as discs with diameter of 2 cm and tested with a gap distance of 1 mm. The viscosity changes were recorded with different shear rates in a range of 0.01–100 s^−1^. The storage (G′) and loss (G″) moduli with increasing shear strength from 0.1 to 10,000 Pa were tested by using oscillatory stress sweep experiments s at a fixed frequency of 1 Hz. The yield strength of hydrogels can be read at the crossover point of G′ and G. The self-healing assay was tested as follows: hydrogel sample was cut off and combined again at room temperature. After 30 min, the conductivity of healed sample was tested by lighting a lamp.

### 2.5. Cell Study

ADA, gelatin and MXenes were sterilized under UV light for 2 h before use. The hydrogel samples were prepared in 12-well plates on a sterilized bench. It should be noted that 0.1% of CaCl_2_ and 2.5% of mTG solution was used as post-cross-linking solution for all samples. Then, the as-prepared hydrogel discs were immersed in cell culture medium containing 89% Dulbecco’s Modified Eagle’s Medium (DMEM, Solarbio Co., Ltd., Beijing, China), 10% fetal bovine serum (FBS, Gibco|Thermo Fisher Scientific^TM^, Waltham, MA, USA) and 1% antibiotic/antimycotic (Solarbio Co., Ltd., China) for 24 h in an incubator at 37 °C with 5% CO_2_. On the second day, the samples were taken out and washed with DPBS twice. After that, NH3T3s were seeded on the surface of each sample disc at the density of 5 × 10^5^ cells per well, followed by incubation with 600 μL of cell culture medium for 6 h at 37 °C with 5% CO_2_. Then, more medium was added to each well to 2 mL and exchanged every 2 days. 

Live-dead staining assay was carried out after a one-day culture. Every sample was washed with DPBS solution and stained with a mixture solution containing Calcein AM (2 μM) and ethidium homodimer1 (2 μM) in DPBS. Images of stained cells were recorded by a fluorescent confocal microscope (Nikon, Tokyo, Japan). Cell Counting Kit-8 (CCK-8) assays were applied to the tripartite samples to evaluate the cell viability. Cell samples were incubated in 1% CCK-8 solutions for 3 h in incubator and then the absorbance of the supernatant was read their absorbance at 450 nm using a well plate reader (Thermo Fisher Scientific, USA). After a one-week culture, rhodamine Phalloidin and DAPI were applied to stain the F-actin and nuclei of cells.

### 2.6. Data Analysis

The data were expressed as mean ± standard deviation. Statistical significance was assessed using the two-way ANOVA on GraphPad software and the values were considered significant at * *p* < 0.05.

## 3. Results and Discussion

### 3.1. Preparation and Micro-Structures of MXene-ADA-GEL Hydrogels

**Figure 2:**
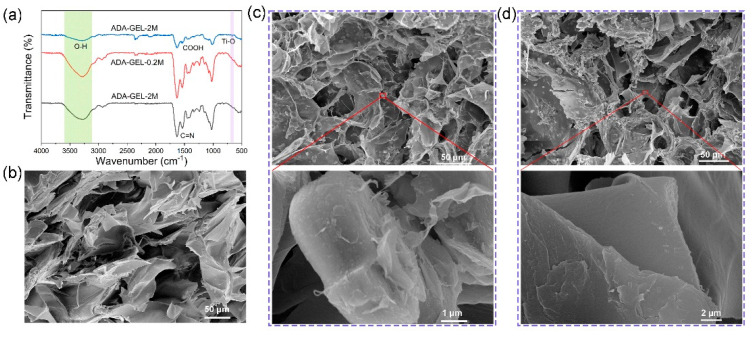
(**a**) FTIR spectra of lyophilized ADA-GEL, ADA-GEL-0.2M and ADA-GEL-2M dry samples. SEM images of the freeze-dried (**b**) ADA-GEL, (**c**) ADA-GEL-0.2M and (**d**) ADA-GEL-2M.

MXene-ADA-GEL hydrogels were prepared by a “one-pot” approach. The uniform MXene/ADA mixture was obtained by adding the well-dispersed MXene solution into the ADA aqueous solution under continuous stirring (see the Materials and Methods Section for details). Then, the gelatin solution was transferred into the mixture of MXene and ADA, and the composite hydrogel was obtained by gelatinization. Figure 1a illustrates the chemical mechanisms for synthesizing the MXene-ADA-GEL where it can be seen that fast gelation takes place benefiting from the interactions among the abundant surface-terminated groups of MXene nanosheets and the polymer chains, as well as the imine bonds of the aldehyde groups from ADA and the amino groups from gelatin. The chemical interactions were analyzed by Fourier transform infrared (FTIR) spectroscopy (Figure 2a). For pure ADA-GEL, the peak shifts that are shown at 1522 cm^−1^ (amide II) and 1628 cm^−1^ (amide I) are attributed to the Schiff base reaction between ADA and GEL [24,27]. The typical peaks of MXene are located at 3420, 1095, and 584 cm^−1^, corresponding to –OH, C–F, and Ti–O, respectively [29]. It can be clearly seen that Ti–O peaks appeared on the FTIR spectrum of MXene-ADA-GEL with an MXene content of 2 *w*/*v*%. Moreover, The O–H stretching band of the MXene-ADA-GEL shifts to a higher wavenumber compared with pure ADA-GEL, demonstrating the presence of hydrogen bonds between MXene and the polymer network. In addition, the freeze-dried hydrogels were observed by SEM to analyze their microstructures. The original MXene flakes show multi-layered sandwich structures (Figure 1), as reported previously [30]. A loose and porous structure was observed on the ADA-GEL, indicating the imine interactions among the polymer chains in Figure 2b. Figure 2c,d shows SEM images of the MXene-ADA-GELs and the pore size is evidently smaller than that of the pure ADA-GEL which may be owing to the stronger interactions of the hybrid network. As a result, the swelling ratio decreased in the hydrogels containing MXenes due to the denser network (Figure S1). Some defects and mild degradation are visible on the surface of the MXene nano-flakes, which could be a result of partial oxidation of MXenes [30]. 

### 3.2. Characterizations of the Rheology, Self-Healing Property, Extrusion Capacity and Electroactivity of MXene-ADA-GEL Hydrogels

**Figure 3:**
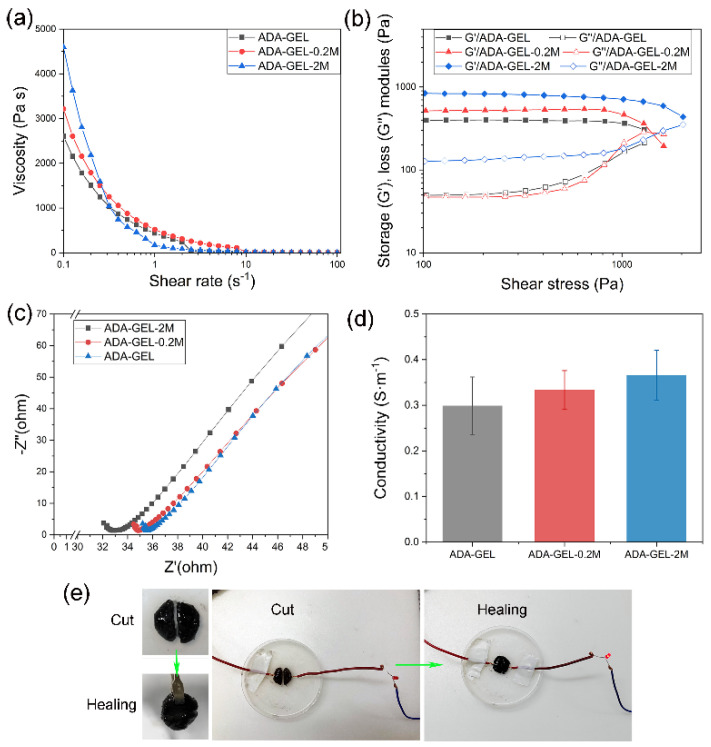
(**a**) Variations of viscosities with increasing shear rate and (**b**) variations of storage and loss moduli with shear stress of different hydrogels. (**c**) The Nyquist plot and (**d**) conductivity of different hydrogels. (**e**) The self-healing and the ability of MXene-ADA-GEL with a light lamp.

The effect of MXene addition on the rheological properties of ADA-GEL was investigated in Figure 3a,b. Figure 3a shows the viscosity of various hydrogels at a shear rate ranging from 0.1 to 100 s^−1^. All samples exhibit shear thinning properties, specifically, the viscosity increases with increasing MXene proportion at a low shear rate (0.1 s^−1^). Afterward, the storage and loss modules of all hydrogels were tested with increasing shear stress in Figure 3b. The storage module increased from 393 ± 12 Pa to 895 ± 14 Pa and the yield strength increased from 1329 ± 21 Pa to 2067 ± 17 Pa with increasing MXene content from 0 to 2 *w*/*v*%. The mechanism of mechanical enhancement could be a result of the more extensive interactions of the composite hydrogel network with the incorporation of MXene nano-flakes. Despite the imine linkages of ADA and gelatin, Mxenes with abundant surface groups were hypothesized to generate hydrogen bonds with the active groups of ADA and gelatin such as –COOH, –NH_2_ and –OH, and this enables the formation of the double cross-linking network inside of the MXene-ADA-GELs, which may play a key role in the mechanical strengthening [31,32,33]. The above results indicate that MXene as a nano-filler can dramatically enhance the mechanical properties of nanocomposite hydrogels owing to a dual-network structure, which was in agreement with the SEM observations and previous reports about MXene-based hybrid hydrogels [34,35]. 

The conductivity of various hydrogels was analyzed by testing their alternating current impedance using a two-electrode system on an electrochemical workstation. The Nyquist curve obtained from Electrochemical Impedance Spectroscopy (EIS) is displayed in Figure 3c. As compared with pure ADA-GEL, the impedance decreased while improving the MXene content inside the hydrogel network as expected, however, without significant difference. Then, the hydrogel conductivity was calculated, as shown in Figure 3d, and the conductivity increased from 0.299 ± 0.063 S m^−1^ to 0.366 ± 0.055 S m^−1^ when 2 *w*/*v*% of MXenes were added into the ADA-GEL. In addition, those MXene-ADA-GELs exhibit superior self-healing properties, as shown in Figure 3e. To better reveal the conductivity and self-healing ability, cut ADA-GEL-0.2M was connected to a lamp bulb powered by an input voltage of three volts. The lamp was lightened when putting the two halves of ADA-GEL-0.2M together, demonstrating the well-performed conductivity and healing properties. Figure S2 displayed the superior self-healing properties of all groups as well. Therefore, it can be concluded that the MXene-ADA-GEL is a good candidate for bioelectronic applications. The ADA-GEL-0.2M was able to self-heal without any external stimulus under room temperature for half an hour after cutting using a bistoury. 

### 3.3. Cell Study

**Figure 4:**
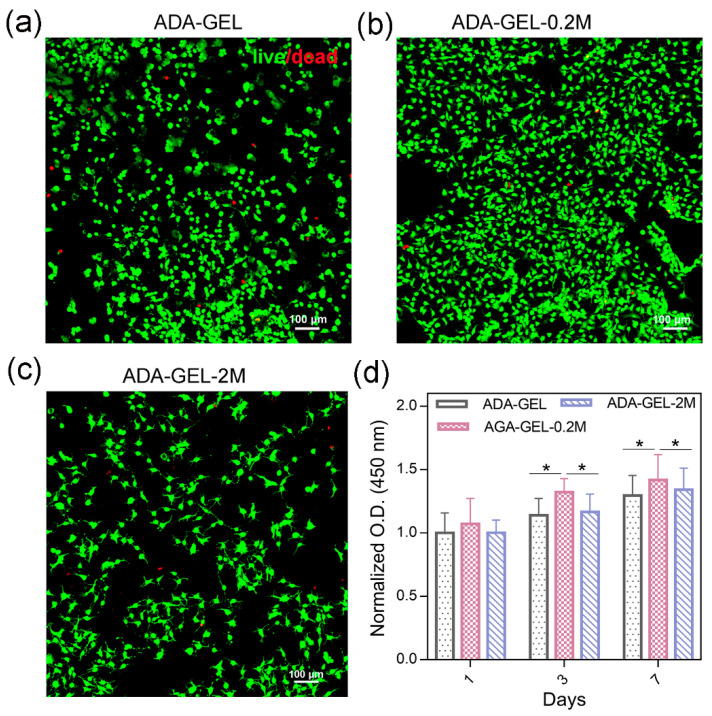
(**a**–**c**) LIVE (green)/ DEAD (red) cell staining of NH3T3s on (**a**) ADA-GEL, (**b**) ADA-GEL-0.2M and (**c**) ADA-GEL-2M. (**d**) NH3T3 viability cultured on different hydrogels for 1 week tested by CCK-8 assay. Statistically significant differences are indicated as: * *p* < 0.05, n = 3.

Confocal images were taken after the live/dead staining of cells, as shown in Figure 4a–c. NH3T3 mouse fibroblast cells grew normally when seeded on different types of hydrogels after day 1, indicating that the MN-MXenes-ADA-GELs were not cytotoxic. Interestingly, most of the NH3T3s are round or elliptical on the ADA-GEL surface, while NH3T3s are more spindle-shaped and have extended pseudopods in the MXene-incorporated hydrogels. Most cells show clear pseudopodium in the ADA-GEL-2M, which is a sign of good cell communication (Figure 4c), suggesting that the MXene composite ADA-GEL can promote cell spreading and attachment. It is hypothesized that the high charge transfer efficiency of MXene stimulates intercellular communication, and the abundant attaching sites of MXenes promote cell adhesion. A CCK8 assay was performed to measure NH3T3 viability after 7 days of incubation seeded on hydrogel discs, as shown in Figure 4d, and the pure ADA-GEL was used as the control. The introduction of MXenes at 0.2 *w*/*v*% appeared to increase the cell viability compared to pure ADA-GEL and with significance on days 3 and 7 (* *p* < 0.05), while the 2 *w*/*v*% of MXenes slightly restrained cell proliferation, however, without significant difference compared to the pure ADA-GEL. It should also be noted that cells on all samples show proliferative trends with a one-week incubation.

**Figure 5:**
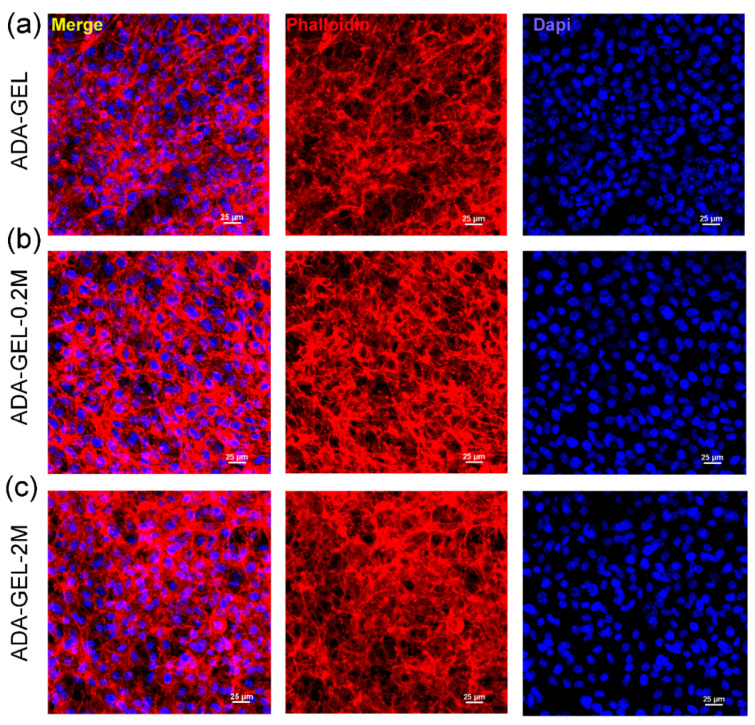
Fluorescence microscopy images of NH3T3s stained with F-actin (red) and cell nuclei (blue) after incubation for 7 days on (**a**) ADA-GELs, (**b**) ADA-GEL-0.2M and (**c**) ADA-GEL-2M.

Actin filaments (F-actin) and DAPI staining were performed to determine the morphology of the cultured NH3T3s on different hydrogels (Figure 5). After 7 days of culture, the cells extended connections with the neighboring cells and formed a dense cell network, indicating the superior spreading ability of all hydrogels. The NH3T3s seeded on the MXene-based ADA-GELs grew in clusters even more tightly connected to the neighboring cells, indicating the better suitability of composited ADA-GELs for NH3T3s in comparison to the MXene-free ADA-GELs. This finding is in consistent with previous research that showed better cell spreading and migration behaviors in MXene-involved hydrogels due to the more hydrophilic MXene built-up flakes present in the heterostructure [36,37,38,39]. Taken as a whole, the above results indicate the superior biocompatibility of MXene-ADA-GELs, which could afford a beneficial micro-environment for cell growth and spreading.

## 4. Conclusions

In summary, a new class of self-healable, electroactive and biocompatible MXene incorporated ADA-GELs was developed as a potential candidate for multifunctional E-skins and acceleration of skin wound recovery. The introduction of MXene nano-flakes has an enhancing effect on polymer cross-linking networks owing to the abundant surface functional groups (–OH, –F, –O, etc.) on the surface of the MXene nano-flakes, resulting in increased viscosity, modulus and yield stress of the composite ADA-GELs. Meanwhile, the MXene-ADA-GELs exhibited good self-healing capability as well as reliable elasticity, showing promising capacity as a wearable sensor to monitor the electrophysiological signals of user movements. Particularly, in the presence of MXenes, the composite hydrogels can promote the spreading and communication of NH3T3 cells in vitro as evidenced by the live-dead and F-actin staining images. We expect that the developed MXene-ADA-GELs can pave the way for prospective applications of MXene-based conducting hydrogels in wearable soft electronics and healthcare monitoring fields.

## Data Availability

The data presented in this study are available from the listed authors.

## Supplementary Materials

The following supporting information can be downloaded at: https://www.mdpi.com/2073-4360/14/18/3908/s1, Figure S1: The swelling ratio of different hydrogels immersed in DPBS solution at 37 °C for up to 8 h; Figure S2: The self-healing behavior of different hydrogels.
