# Correction: Zhu et al. Electroactive Oxidized Alginate/Gelatin/MXene (Ti_3_C_2_Tx) Composite Hydrogel with Improved Biocompatibility and Self-Healing Property. *Polymers* 2022, *14*, 3908

**DOI:** 10.3390/polym14235212

**Published:** 2022-11-30

**Authors:** Hui Zhu, Weitao Dai, Liming Wang, Cong Yao, Chenxi Wang, Bingsong Gu, Dichen Li, Jiankang He

**Affiliations:** 1 State Key Laboratory for Manufacturing Systems Engineering, Xi’an Jiaotong University, Xi’an 710049, China; 2 NMPA Key Laboratory for Research and Evaluation of Additive Manufacturing Medical Devices, Xi’an Jiaotong University, Xi’an 710049, China

In the original publication [1], there were mistakes in Figure 3d and the relevant description as published. There was an error regarding the y-coordinate of Figure 3d, meaning that the value of conductivity was wrong. The corrected Figure 3 and the relevant description appear below.

**Figure 3:**
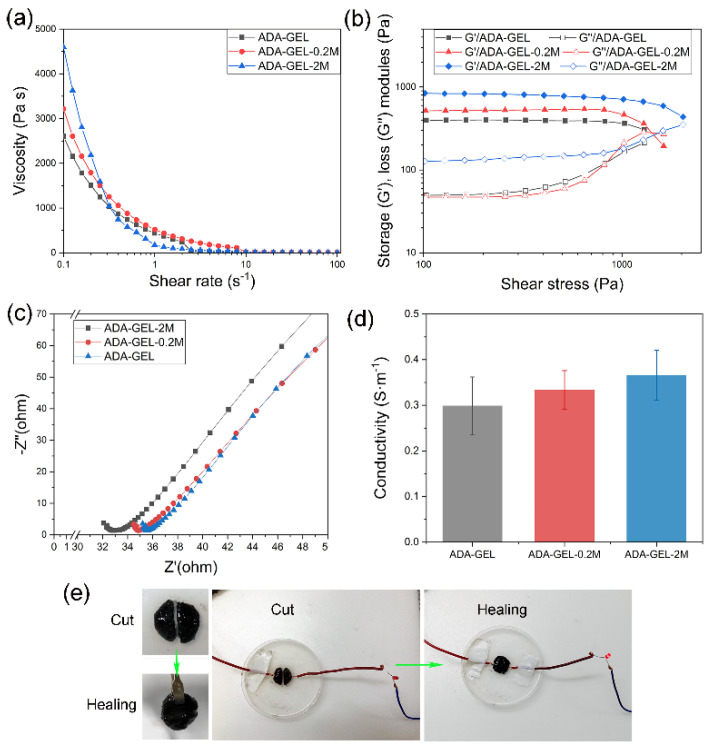
(**a**) Variations of viscosities with increasing shear rate and (**b**) variations of storage and loss moduli with shear stress of different hydrogels. (**c**) The Nyquist plot and (**d**) conductivity of different hydrogels. (**e**) The self-healing and the ability of MXene-ADA-GEL with a light lamp.

In Section 3.2, paragraph 2: “…and the conductivity increased from 2.99 ± 0.63 S m^−1^ to 3.66 ± 0.55 S m^−1^ when 2 *w*/*v*% of MXenes were added into the ADA-GEL” should be changed to “…and the conductivity increased from 0.299 ± 0.063 S m^−1^ to 0.366 ± 0.055 S m^−1^ when 2 *w*/*v*% of MXenes were added into the ADA-GEL”.

The authors state that the scientific conclusions are unaffected. This correction was approved by the Academic Editor. The original publication has also been updated.
