# Supplementary material for: Electroactive Oxidized Alginate/Gelatin/MXene (Ti3C2Tx) Composite Hydrogel with Improved Biocompatibility and Self-Healing Property
Source: Polymers (Basel). 2022 Sep 19;14(18):3908. doi: 10.3390/polym14183908 (PMC9506128; doi:10.3390/polym14183908)
Supplement: Supplementary file 1 [file polymers-14-03908-s001.zip › polymers-1890707-supplementary.pdf]

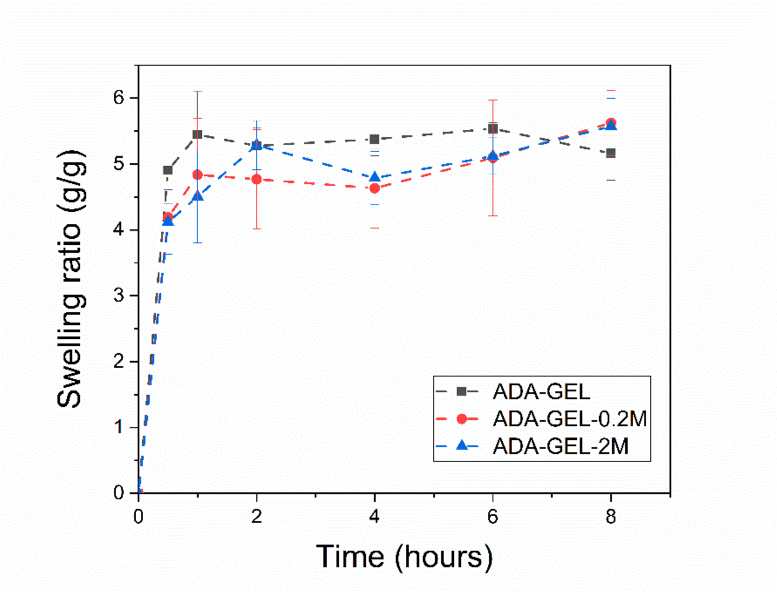

Figure S1. The swelling ratio of different hydrogels immersed in DPBS solution at 37°C for up to 8 hours.

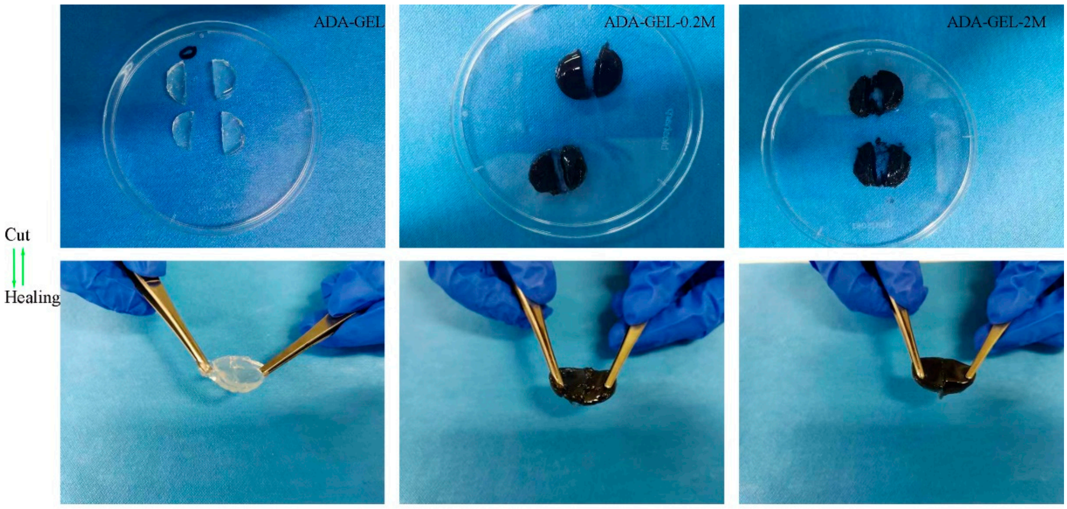

Figure S2. The self-healing behavior of different hydrogels
